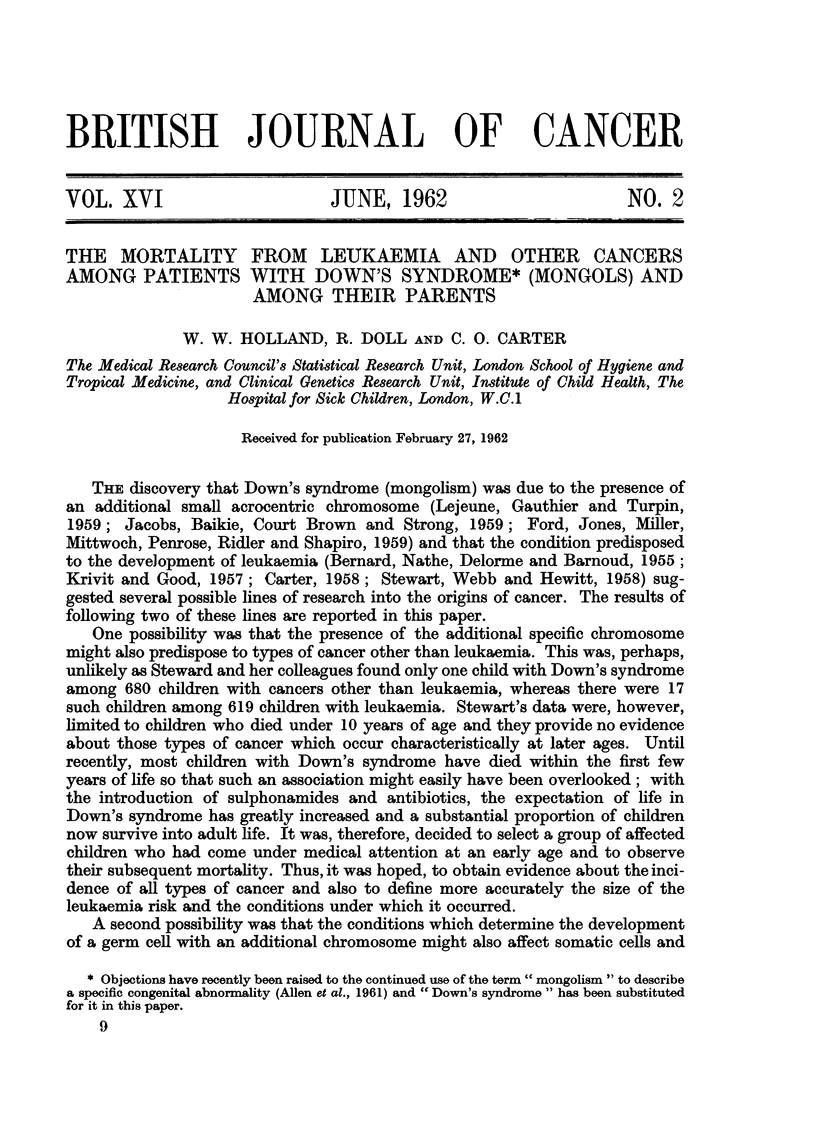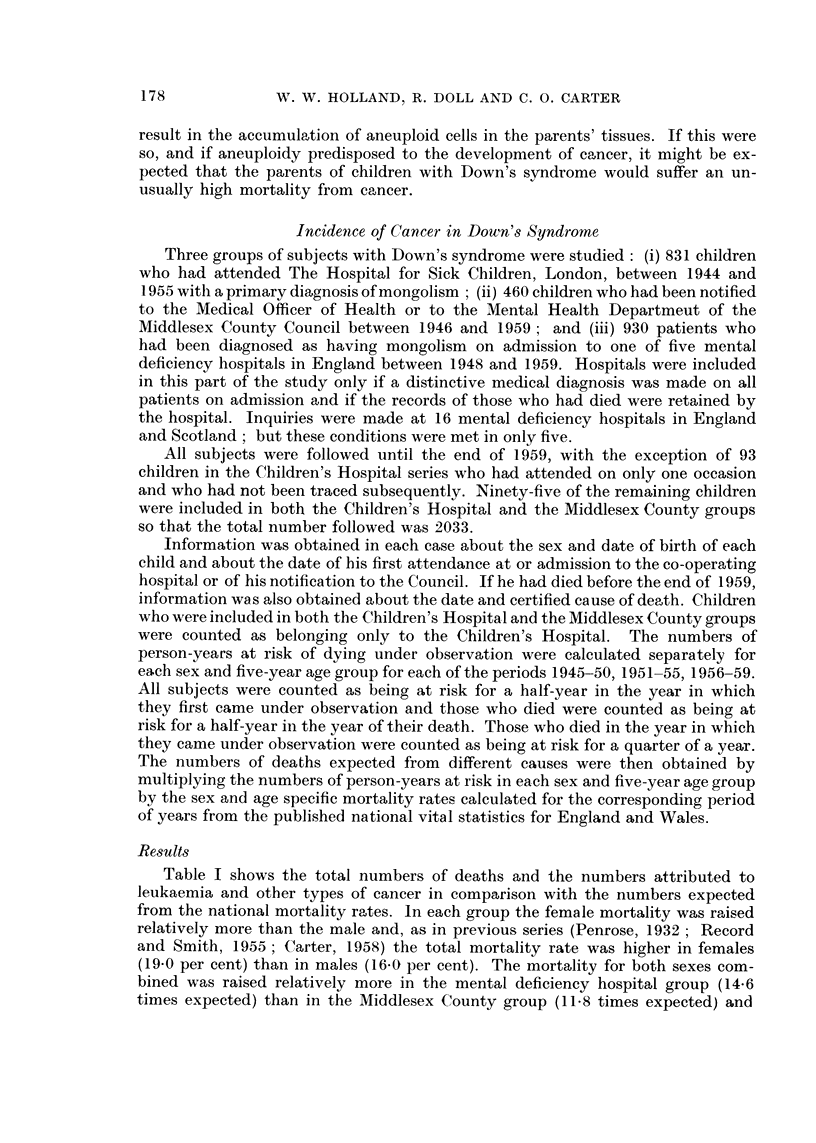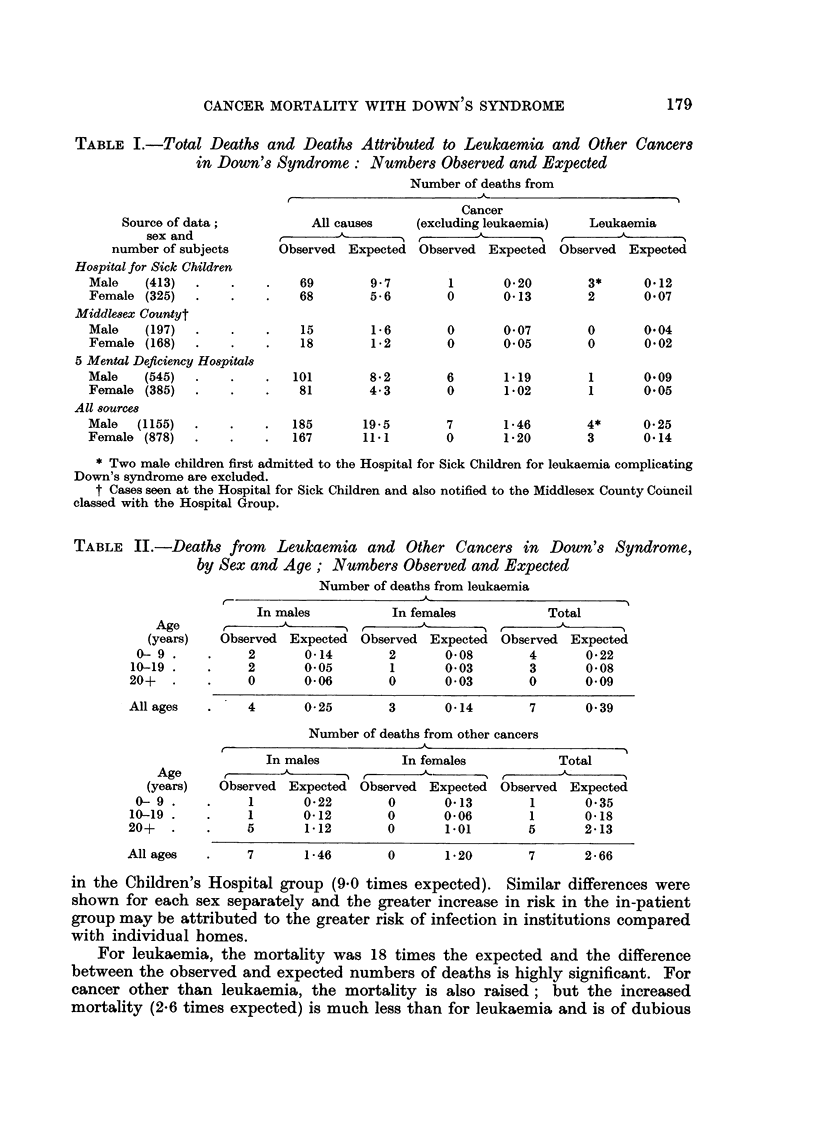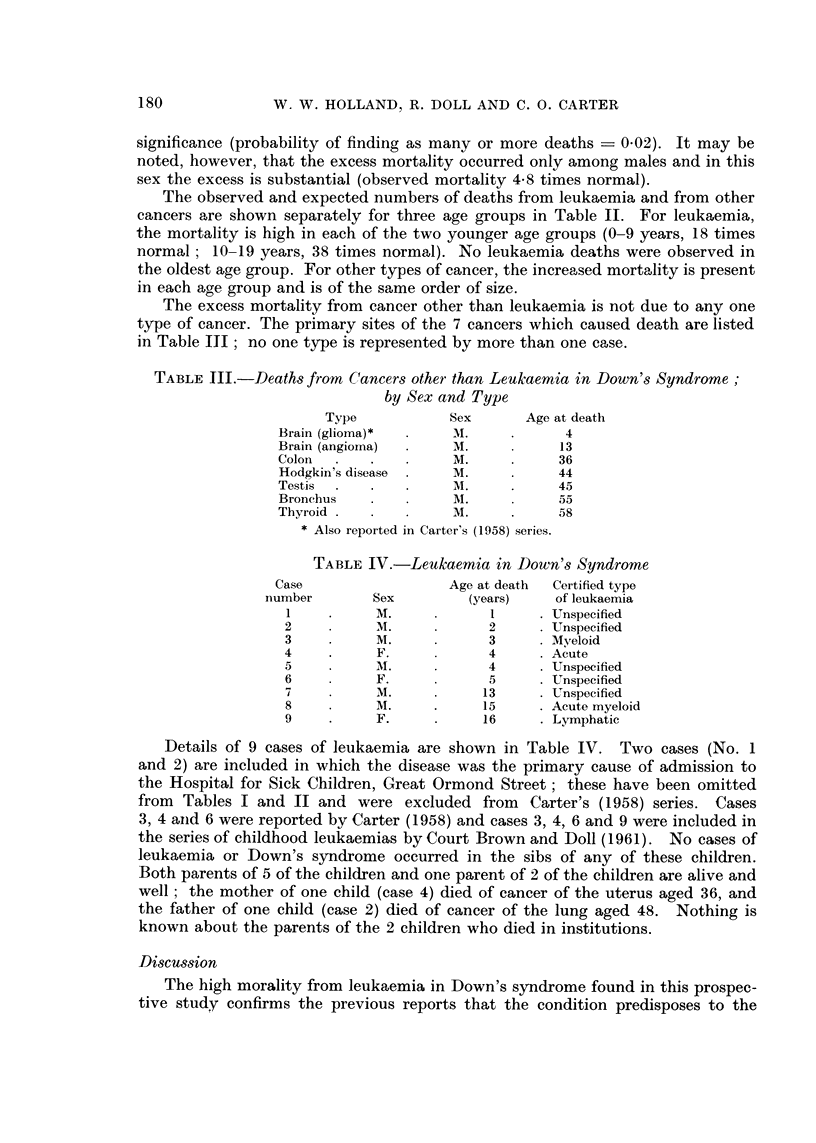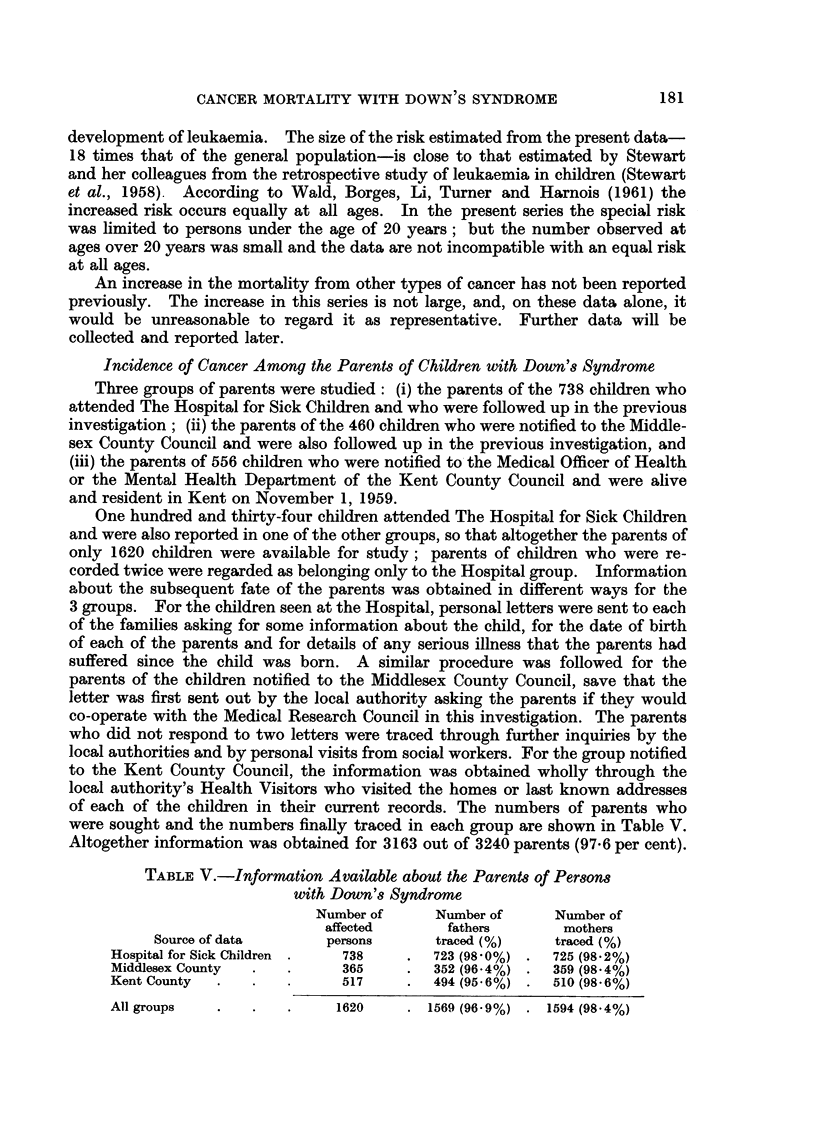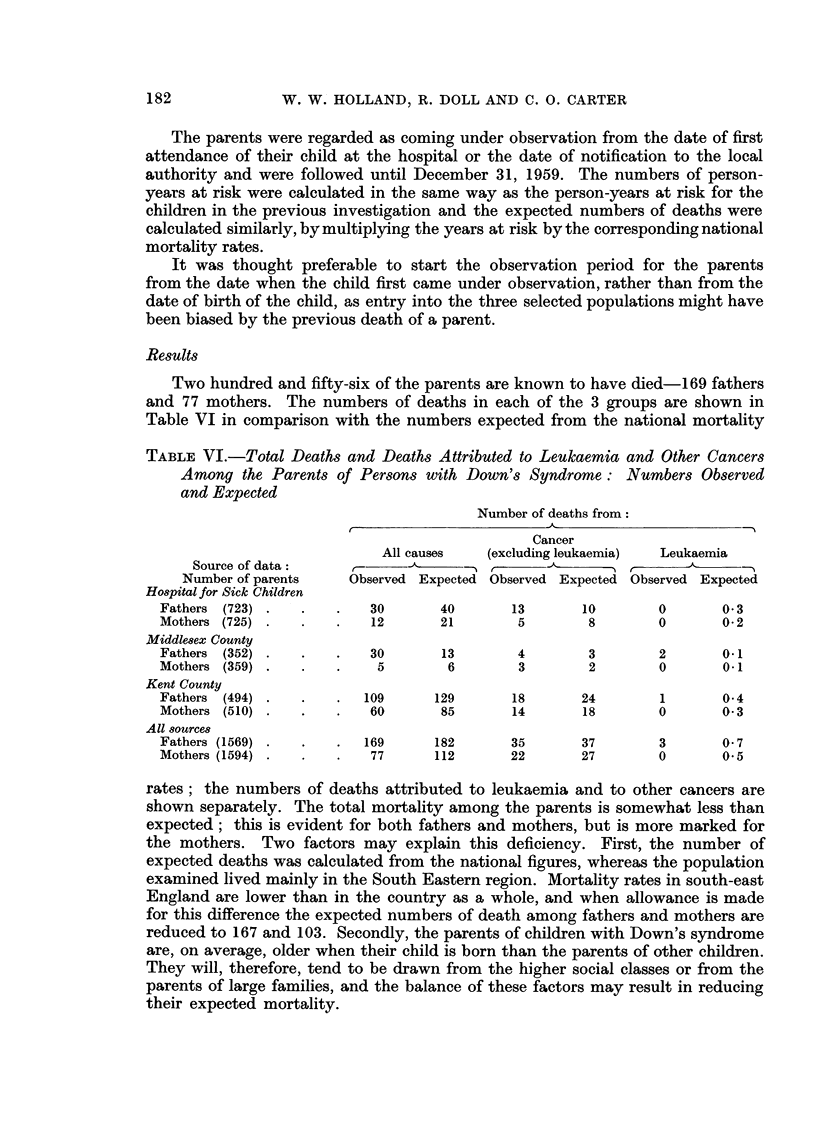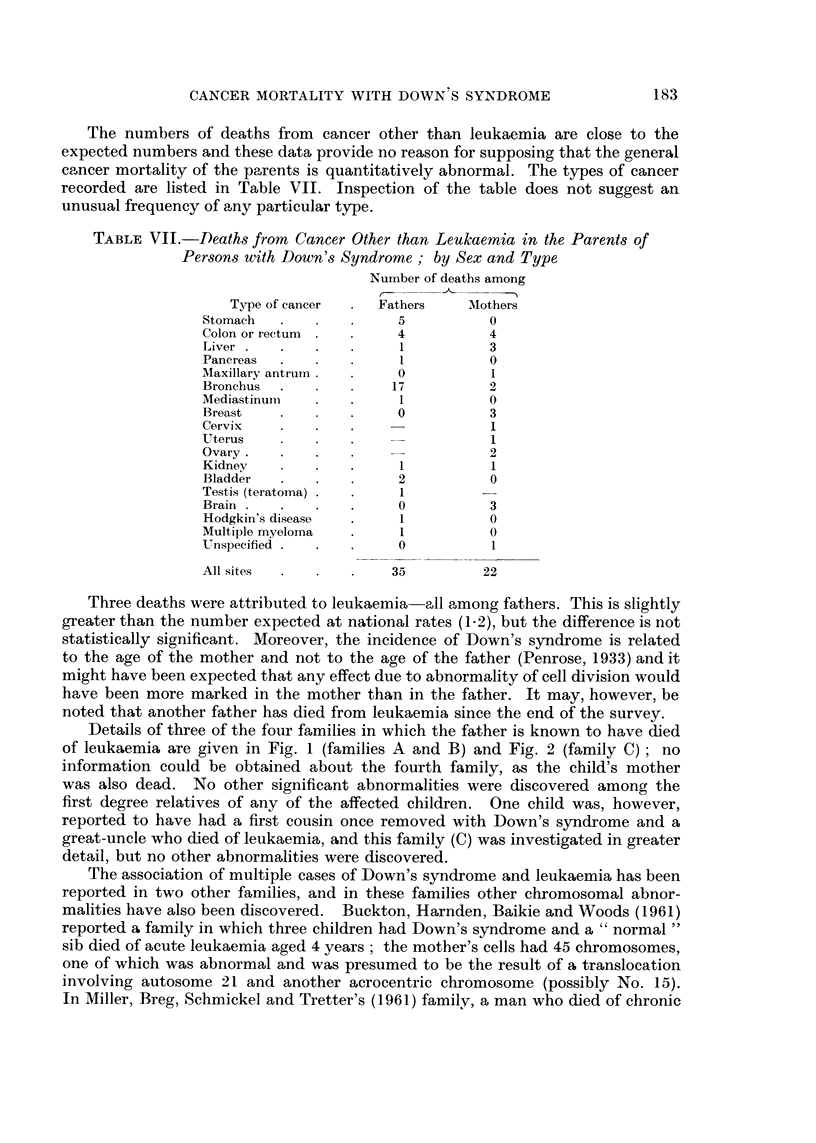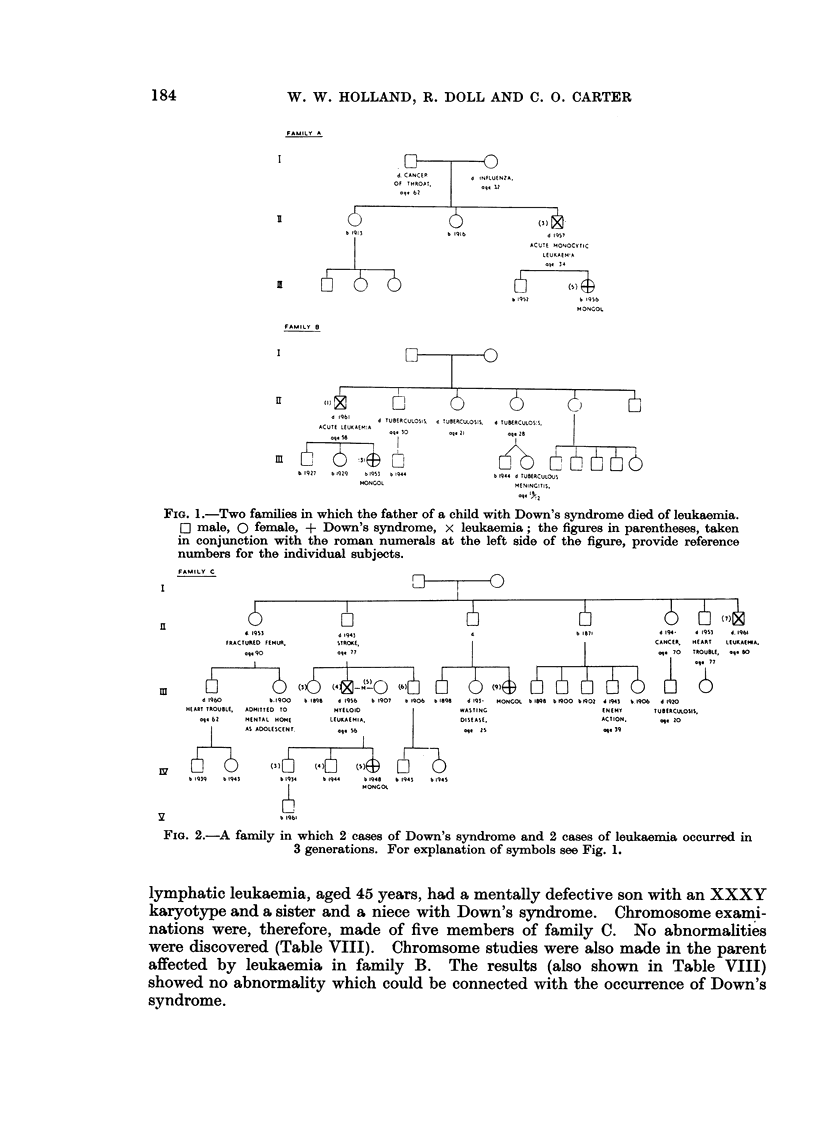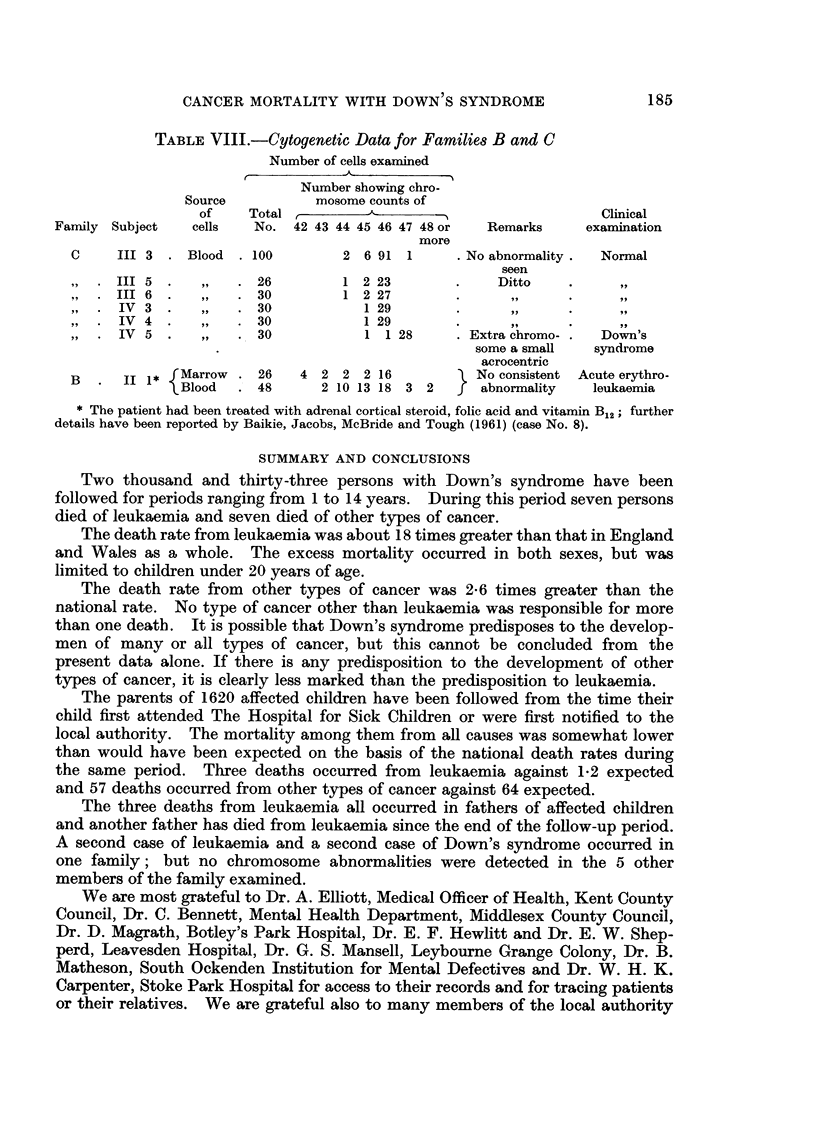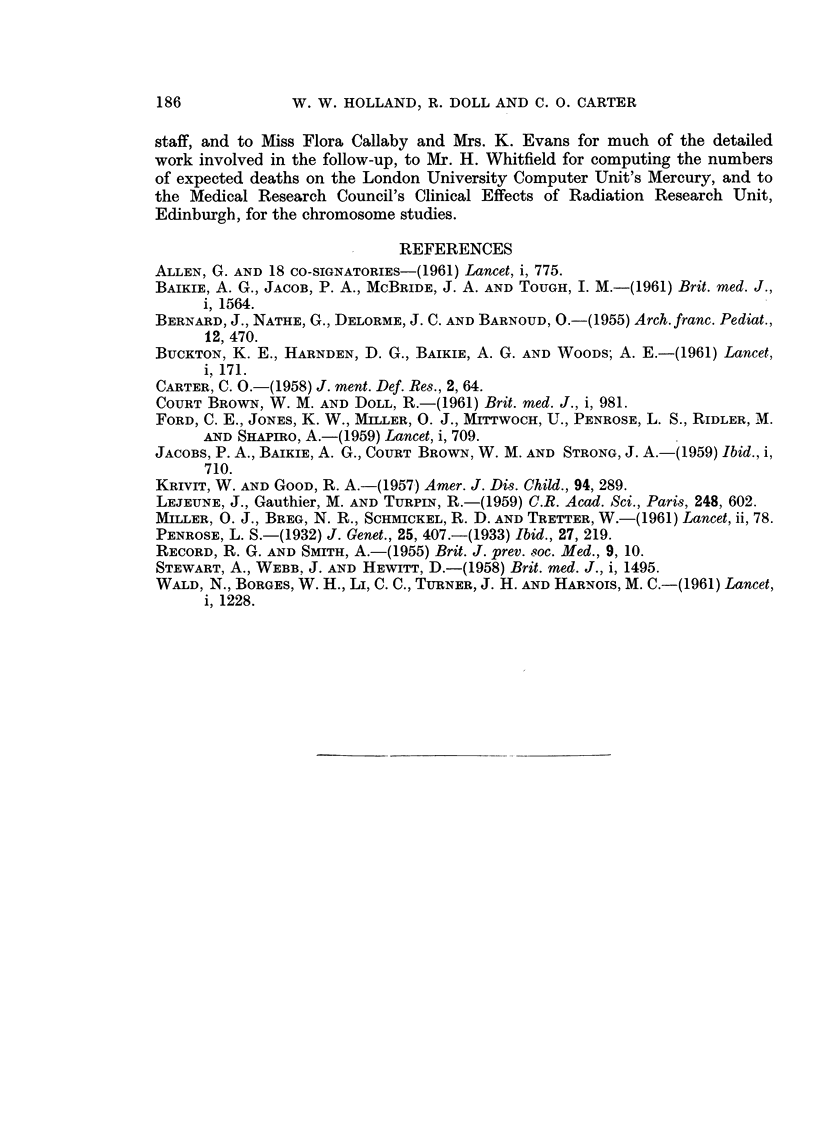# The Mortality from Leukaemia and Other Cancers Among Patients with Down's Syndrome (Mongols) and Among their Parents*

**DOI:** 10.1038/bjc.1962.20

**Published:** 1962-06

**Authors:** W. W. Holland, R. Doll, C. O. Carter


// BRITISH JOURNAL OF CANCER

VOL. XVI JUNE, 1962 NO. 2

### THE MORTALITY FROM LEUKAEMIA AND OTHER CANCERS AMONG PATIENTS WITH DOWN'S SYNDROME* (MONGOLS) AND AMONG THEIR PARENTS

W. W. HOLLAND, R. DOLL AND C. O. CARTER

*The Medical Research Council's Statistical Research Unit, London School of Hygiene and Tropical Medicine, and Clinical Genetics Research Unit, Institute of Child Health, The Hospital for Sick Children, London, W.C.1*

Received for publication February 27, 1962

THE discovery that Down's syndrome (mongolism) was due to the presence of an additional small acrocentric chromosome (Lejeune, Gauthier and Turpin, 1959; Jacobs, Baikie, Court Brown and Strong, 1959; Ford, Jones, Miller, Mittwoch, Penrose, Ridler and Shapiro, 1959) and that the condition predisposed to the development of leukaemia (Bernard, Nathe, Delorme and Barnoud, 1955; Krivit and Good, 1957; Carter, 1958; Stewart, Webb and Hewitt, 1958) suggested several possible lines of research into the origins of cancer. The results of following two of these lines are reported in this paper.

One possibility was that the presence of the additional specific chromosome might also predispose to types of cancer other than leukaemia. This was, perhaps, unlikely as Steward and her colleagues found only one child with Down's syndrome among 680 children with cancers other than leukaemia, whereas there were 17 such children among 619 children with leukaemia. Stewart's data were, however, limited to children who died under 10 years of age and they provide no evidence about those types of cancer which occur characteristically at later ages. Until recently, most children with Down's syndrome have died within the first few years of life so that such an association might easily have been overlooked; with the introduction of sulphonamides and antibiotics, the expectation of life in Down's syndrome has greatly increased and a substantial proportion of children now survive into adult life. It was, therefore, decided to select a group of affected children who had come under medical attention at an early age and to observe their subsequent mortality. Thus, it was hoped, to obtain evidence about the incidence of all types of cancer and also to define more accurately the size of the leukaemia risk and the conditions under which it occurred.

A second possibility was that the conditions which determine the development of a germ cell with an additional chromosome might also affect somatic cells and

* Objections have recently been raised to the continued use of the term " mongolism " to describe a specific congenital abnormality (Allen *et al.*, 1961) and " Down's syndrome " has been substituted for it in this paper.

result in the accumulation of aneuploid cells in the parents' tissues. If this were so, and if aneuploidy predisposed to the development of cancer, it might be expected that the parents of children with Down's syndrome would suffer an unusually high mortality from cancer.

### *Incidence of Cancer in Down's Syndrome*

Three groups of subjects with Down's syndrome were studied : (i) 831 children who had attended The Hospital for Sick Children, London, between 1944 and 1955 with a primary diagnosis of mongolism ; (ii) 460 children who had been notified to the Medical Officer of Health or to the Mental Health Departmeut of the Middlesex County Council between 1946 and 1959 ; and (iii) 930 patients who had been diagnosed as having mongolism on admission to one of five mental deficiency hospitals in England between 1948 and 1959. Hospitals were included in this part of the study only if a distinctive medical diagnosis was made on all patients on admission and if the records of those who had died were retained by the hospital. Inquiries were made at 16 mental deficiency hospitals in England and Scotland ; but these conditions were met in only five.

All subjects were followed until the end of 1959, with the exception of 93 children in the Children's Hospital series who had attended on only one occasion and who had not been traced subsequently. Ninety-five of the remaining children were included in both the Children's Hospital and the Middlesex County groups so that the total number followed was 2033.

Information was obtained in each case about the sex and date of birth of each child and about the date of his first attendance at or admission to the co-operating hospital or of his notification to the Council. If he had died before the end of 1959, information was also obtained about the date and certified cause of death. Children who were included in both the Children's Hospital and the Middlesex County groups were counted as belonging only to the Children's Hospital. The numbers of person-years at risk of dying under observation were calculated separately for each sex and five-year age group for each of the periods 1945–50, 1951–55, 1956–59. All subjects were counted as being at risk for a half-year in the year in which they first came under observation and those who died were counted as being at risk for a half-year in the year of their death. Those who died in the year in which they came under observation were counted as being at risk for a quarter of a year. The numbers of deaths expected from different causes were then obtained by multiplying the numbers of person-years at risk in each sex and five-year age group by the sex and age specific mortality rates calculated for the corresponding period of years from the published national vital statistics for England and Wales.

### *Results*

Table I shows the total numbers of deaths and the numbers attributed to leukaemia and other types of cancer in comparison with the numbers expected from the national mortality rates. In each group the female mortality was raised relatively more than the male and, as in previous series (Penrose, 1932 ; Record and Smith, 1955 ; Carter, 1958) the total mortality rate was higher in females (19·0 per cent) than in males (16·0 per cent). The mortality for both sexes combined was raised relatively more in the mental deficiency hospital group (14·6 times expected) than in the Middlesex County group (11·8 times expected) and

TABLE I.—*Total Deaths and Deaths Attributed to Leukaemia and Other Cancers in Down's Syndrome : Numbers Observed and Expected*

| Source of data ; sex and number of subjects | Number of deaths from: All causes | | Cancer (excluding leukaemia) | | Leukaemia | |
|---|---|---|---|---|---|---|
| | Observed | Expected | Observed | Expected | Observed | Expected |
| *Hospital for Sick Children* | | | | | | |
| Male (413) . . . . | 69 | 9·7 | 1 | 0·20 | 3* | 0·12 |
| Female (325) . . . . | 68 | 5·6 | 0 | 0·13 | 2 | 0·07 |
| *Middlesex County*† | | | | | | |
| Male (197) . . . . | 15 | 1·6 | 0 | 0·07 | 0 | 0·04 |
| Female (168) . . . . | 18 | 1·2 | 0 | 0·05 | 0 | 0·02 |
| *5 Mental Deficiency Hospitals* | | | | | | |
| Male (545) . . . . | 101 | 8·2 | 6 | 1·19 | 1 | 0·09 |
| Female (385) . . . . | 81 | 4·3 | 0 | 1·02 | 1 | 0·05 |
| *All sources* | | | | | | |
| Male (1155) . . . . | 185 | 19·5 | 7 | 1·46 | 4* | 0·25 |
| Female (878) . . . . | 167 | 11·1 | 0 | 1·20 | 3 | 0·14 |

* Two male children first admitted to the Hospital for Sick Children for leukaemia complicating Down's syndrome are excluded.

† Cases seen at the Hospital for Sick Children and also notified to the Middlesex County Council classed with the Hospital Group.

TABLE II.—*Deaths from Leukaemia and Other Cancers in Down's Syndrome, by Sex and Age ; Numbers Observed and Expected*

| Age (years) | Number of deaths from leukaemia: In males | | In females | | Total | |
|---|---|---|---|---|---|---|
| | Observed | Expected | Observed | Expected | Observed | Expected |
| 0– 9 . . | 2 | 0·14 | 2 | 0·08 | 4 | 0·22 |
| 10–19 . . | 2 | 0·05 | 1 | 0·03 | 3 | 0·08 |
| 20+ . . | 0 | 0·06 | 0 | 0·03 | 0 | 0·09 |
| All ages . | 4 | 0·25 | 3 | 0·14 | 7 | 0·39 |

| Age (years) | Number of deaths from other cancers: In males | | In females | | Total | |
|---|---|---|---|---|---|---|
| | Observed | Expected | Observed | Expected | Observed | Expected |
| 0– 9 . . | 1 | 0·22 | 0 | 0·13 | 1 | 0·35 |
| 10–19 . . | 1 | 0·12 | 0 | 0·06 | 1 | 0·18 |
| 20+ . . | 5 | 1·12 | 0 | 1·01 | 5 | 2·13 |
| All ages . | 7 | 1·46 | 0 | 1·20 | 7 | 2·66 |

in the Children's Hospital group (9·0 times expected). Similar differences were shown for each sex separately and the greater increase in risk in the in-patient group may be attributed to the greater risk of infection in institutions compared with individual homes.

For leukaemia, the mortality was 18 times the expected and the difference between the observed and expected numbers of deaths is highly significant. For cancer other than leukaemia, the mortality is also raised ; but the increased mortality (2·6 times expected) is much less than for leukaemia and is of dubious significance (probability of finding as many or more deaths = 0·02). It may be noted, however, that the excess mortality occurred only among males and in this sex the excess is substantial (observed mortality 4·8 times normal).

The observed and expected numbers of deaths from leukaemia and from other cancers are shown separately for three age groups in Table II. For leukaemia, the mortality is high in each of the two younger age groups (0–9 years, 18 times normal ; 10–19 years, 38 times normal). No leukaemia deaths were observed in the oldest age group. For other types of cancer, the increased mortality is present in each age group and is of the same order of size.

The excess mortality from cancer other than leukaemia is not due to any one type of cancer. The primary sites of the 7 cancers which caused death are listed in Table III ; no one type is represented by more than one case.

TABLE III.—*Deaths from Cancers other than Leukaemia in Down's Syndrome ; by Sex and Type*

| Type | Sex | Age at death |
|---|---|---|
| Brain (glioma)* | M. | 4 |
| Brain (angioma) | M. | 13 |
| Colon | M. | 36 |
| Hodgkin's disease | M. | 44 |
| Testis | M. | 45 |
| Bronchus | M. | 55 |
| Thyroid | M. | 58 |

* Also reported in Carter's (1958) series.

TABLE IV.—*Leukaemia in Down's Syndrome*

| Case number | Sex | Age at death (years) | Certified type of leukaemia |
|---|---|---|---|
| 1 | M. | 1 | Unspecified |
| 2 | M. | 2 | Unspecified |
| 3 | M. | 3 | Myeloid |
| 4 | F. | 4 | Acute |
| 5 | M. | 4 | Unspecified |
| 6 | F. | 5 | Unspecified |
| 7 | M. | 13 | Unspecified |
| 8 | M. | 15 | Acute myeloid |
| 9 | F. | 16 | Lymphatic |

Details of 9 cases of leukaemia are shown in Table IV. Two cases (No. 1 and 2) are included in which the disease was the primary cause of admission to the Hospital for Sick Children, Great Ormond Street ; these have been omitted from Tables I and II and were excluded from Carter's (1958) series. Cases 3, 4 and 6 were reported by Carter (1958) and cases 3, 4, 6 and 9 were included in the series of childhood leukaemias by Court Brown and Doll (1961). No cases of leukaemia or Down's syndrome occurred in the sibs of any of these children. Both parents of 5 of the children and one parent of 2 of the children are alive and well ; the mother of one child (case 4) died of cancer of the uterus aged 36, and the father of one child (case 2) died of cancer of the lung aged 48. Nothing is known about the parents of the 2 children who died in institutions.

*Discussion*

The high morality from leukaemia in Down's syndrome found in this prospective study confirms the previous reports that the condition predisposes to the development of leukaemia. The size of the risk estimated from the present data—18 times that of the general population—is close to that estimated by Stewart and her colleagues from the retrospective study of leukaemia in children (Stewart *et al.*, 1958). According to Wald, Borges, Li, Turner and Harnois (1961) the increased risk occurs equally at all ages. In the present series the special risk was limited to persons under the age of 20 years ; but the number observed at ages over 20 years was small and the data are not incompatible with an equal risk at all ages.

An increase in the mortality from other types of cancer has not been reported previously. The increase in this series is not large, and, on these data alone, it would be unreasonable to regard it as representative. Further data will be collected and reported later.

### *Incidence of Cancer Among the Parents of Children with Down's Syndrome*

Three groups of parents were studied : (i) the parents of the 738 children who attended The Hospital for Sick Children and who were followed up in the previous investigation ; (ii) the parents of the 460 children who were notified to the Middlesex County Council and were also followed up in the previous investigation, and (iii) the parents of 556 children who were notified to the Medical Officer of Health or the Mental Health Department of the Kent County Council and were alive and resident in Kent on November 1, 1959.

One hundred and thirty-four children attended The Hospital for Sick Children and were also reported in one of the other groups, so that altogether the parents of only 1620 children were available for study ; parents of children who were recorded twice were regarded as belonging only to the Hospital group. Information about the subsequent fate of the parents was obtained in different ways for the 3 groups. For the children seen at the Hospital, personal letters were sent to each of the families asking for some information about the child, for the date of birth of each of the parents and for details of any serious illness that the parents had suffered since the child was born. A similar procedure was followed for the parents of the children notified to the Middlesex County Council, save that the letter was first sent out by the local authority asking the parents if they would co-operate with the Medical Research Council in this investigation. The parents who did not respond to two letters were traced through further inquiries by the local authorities and by personal visits from social workers. For the group notified to the Kent County Council, the information was obtained wholly through the local authority's Health Visitors who visited the homes or last known addresses of each of the children in their current records. The numbers of parents who were sought and the numbers finally traced in each group are shown in Table V. Altogether information was obtained for 3163 out of 3240 parents (97·6 per cent).

TABLE V.—*Information Available about the Parents of Persons with Down's Syndrome*

| Source of data | Number of affected persons | Number of fathers traced (%) | Number of mothers traced (%) |
|---|---|---|---|
| Hospital for Sick Children | 738 | 723 (98·0%) | 725 (98·2%) |
| Middlesex County | 365 | 352 (96·4%) | 359 (98·4%) |
| Kent County | 517 | 494 (95·6%) | 510 (98·6%) |
| All groups | 1620 | 1569 (96·9%) | 1594 (98·4%) |

The parents were regarded as coming under observation from the date of first attendance of their child at the hospital or the date of notification to the local authority and were followed until December 31, 1959. The numbers of person-years at risk were calculated in the same way as the person-years at risk for the children in the previous investigation and the expected numbers of deaths were calculated similarly, by multiplying the years at risk by the corresponding national mortality rates.

It was thought preferable to start the observation period for the parents from the date when the child first came under observation, rather than from the date of birth of the child, as entry into the three selected populations might have been biased by the previous death of a parent.

*Results*

Two hundred and fifty-six of the parents are known to have died—169 fathers and 77 mothers. The numbers of deaths in each of the 3 groups are shown in Table VI in comparison with the numbers expected from the national mortality

TABLE VI.—*Total Deaths and Deaths Attributed to Leukaemia and Other Cancers Among the Parents of Persons with Down's Syndrome: Numbers Observed and Expected*

| Source of data: Number of parents | Number of deaths from: All causes | | Cancer (excluding leukaemia) | | Leukaemia | |
|---|---|---|---|---|---|---|
| | Observed | Expected | Observed | Expected | Observed | Expected |
| *Hospital for Sick Children* | | | | | | |
| Fathers (723) | 30 | 40 | 13 | 10 | 0 | 0·3 |
| Mothers (725) | 12 | 21 | 5 | 8 | 0 | 0·2 |
| *Middlesex County* | | | | | | |
| Fathers (352) | 30 | 13 | 4 | 3 | 2 | 0·1 |
| Mothers (359) | 5 | 6 | 3 | 2 | 0 | 0·1 |
| *Kent County* | | | | | | |
| Fathers (494) | 109 | 129 | 18 | 24 | 1 | 0·4 |
| Mothers (510) | 60 | 85 | 14 | 18 | 0 | 0·3 |
| *All sources* | | | | | | |
| Fathers (1569) | 169 | 182 | 35 | 37 | 3 | 0·7 |
| Mothers (1594) | 77 | 112 | 22 | 27 | 0 | 0·5 |

rates; the numbers of deaths attributed to leukaemia and to other cancers are shown separately. The total mortality among the parents is somewhat less than expected; this is evident for both fathers and mothers, but is more marked for the mothers. Two factors may explain this deficiency. First, the number of expected deaths was calculated from the national figures, whereas the population examined lived mainly in the South Eastern region. Mortality rates in south-east England are lower than in the country as a whole, and when allowance is made for this difference the expected numbers of death among fathers and mothers are reduced to 167 and 103. Secondly, the parents of children with Down's syndrome are, on average, older when their child is born than the parents of other children. They will, therefore, tend to be drawn from the higher social classes or from the parents of large families, and the balance of these factors may result in reducing their expected mortality.

The numbers of deaths from cancer other than leukaemia are close to the expected numbers and these data provide no reason for supposing that the general cancer mortality of the parents is quantitatively abnormal. The types of cancer recorded are listed in Table VII. Inspection of the table does not suggest an unusual frequency of any particular type.

TABLE VII.—*Deaths from Cancer Other than Leukaemia in the Parents of Persons with Down's Syndrome ; by Sex and Type*

| Type of cancer | Number of deaths among: Fathers | Mothers |
|---|---|---|
| Stomach | 5 | 0 |
| Colon or rectum | 4 | 4 |
| Liver | 1 | 3 |
| Pancreas | 1 | 0 |
| Maxillary antrum | 0 | 1 |
| Bronchus | 17 | 2 |
| Mediastinum | 1 | 0 |
| Breast | 0 | 3 |
| Cervix | — | 1 |
| Uterus | — | 1 |
| Ovary | — | 2 |
| Kidney | 1 | 1 |
| Bladder | 2 | 0 |
| Testis (teratoma) | 1 | — |
| Brain | 0 | 3 |
| Hodgkin's disease | 1 | 0 |
| Multiple myeloma | 1 | 0 |
| Unspecified | 0 | 1 |
| All sites | 35 | 22 |

Three deaths were attributed to leukaemia—all among fathers. This is slightly greater than the number expected at national rates (1·2), but the difference is not statistically significant. Moreover, the incidence of Down's syndrome is related to the age of the mother and not to the age of the father (Penrose, 1933) and it might have been expected that any effect due to abnormality of cell division would have been more marked in the mother than in the father. It may, however, be noted that another father has died from leukaemia since the end of the survey.

Details of three of the four families in which the father is known to have died of leukaemia are given in Fig. 1 (families A and B) and Fig. 2 (family C) ; no information could be obtained about the fourth family, as the child's mother was also dead. No other significant abnormalities were discovered among the first degree relatives of any of the affected children. One child was, however, reported to have had a first cousin once removed with Down's syndrome and a great-uncle who died of leukaemia, and this family (C) was investigated in greater detail, but no other abnormalities were discovered.

The association of multiple cases of Down's syndrome and leukaemia has been reported in two other families, and in these families other chromosomal abnormalities have also been discovered. Buckton, Harnden, Baikie and Woods (1961) reported a family in which three children had Down's syndrome and a " normal " sib died of acute leukaemia aged 4 years ; the mother's cells had 45 chromosomes, one of which was abnormal and was presumed to be the result of a translocation involving autosome 21 and another acrocentric chromosome (possibly No. 15). In Miller, Breg, Schmickel and Tretter's (1961) family, a man who died of chronic

**Fig. 1.**—Two families in which the father of a child with Down's syndrome died of leukaemia. □ male, ○ female, + Down's syndrome, × leukaemia ; the figures in parentheses, taken in conjunction with the roman numerals at the left side of the figure, provide reference numbers for the individual subjects.

**Fig. 2.**—A family in which 2 cases of Down's syndrome and 2 cases of leukaemia occurred in 3 generations. For explanation of symbols see Fig. 1.

lymphatic leukaemia, aged 45 years, had a mentally defective son with an XXXY karyotype and a sister and a niece with Down's syndrome. Chromosome examinations were, therefore, made of five members of family C. No abnormalities were discovered (Table VIII). Chromsome studies were also made in the parent affected by leukaemia in family B. The results (also shown in Table VIII) showed no abnormality which could be connected with the occurrence of Down's syndrome.

TABLE VIII.—*Cytogenetic Data for Families B and C*

| Family | Subject | Source of cells | Number of cells examined: Total No. | Number showing chromosome counts of: 42 | 43 | 44 | 45 | 46 | 47 | 48 or more | Remarks | Clinical examination |
|---|---|---|---|---|---|---|---|---|---|---|---|---|
| C | III 3 | Blood | 100 | | | 2 | 6 | 91 | 1 | | No abnormality seen | Normal |
| ,, | III 5 | ,, | 26 | | | 1 | 2 | 23 | | | Ditto | ,, |
| ,, | III 6 | ,, | 30 | | | 1 | 2 | 27 | | | ,, | ,, |
| ,, | IV 3 | ,, | 30 | | | | 1 | 29 | | | ,, | ,, |
| ,, | IV 4 | ,, | 30 | | | | 1 | 29 | | | ,, | ,, |
| ,, | IV 5 | ,, | 30 | | | | 1 | 1 | 28 | | Extra chromosome a small acrocentric | Down's syndrome |
| B | II 1* | Marrow | 26 | 4 | 2 | 2 | 2 | 16 | | | No consistent abnormality | Acute erythroleukaemia |
| | | Blood | 48 | | 2 | 10 | 13 | 18 | 3 | 2 | | |

* The patient had been treated with adrenal cortical steroid, folic acid and vitamin $B_{12}$; further details have been reported by Baikie, Jacobs, McBride and Tough (1961) (case No. 8).

### SUMMARY AND CONCLUSIONS

Two thousand and thirty-three persons with Down's syndrome have been followed for periods ranging from 1 to 14 years. During this period seven persons died of leukaemia and seven died of other types of cancer.

The death rate from leukaemia was about 18 times greater than that in England and Wales as a whole. The excess mortality occurred in both sexes, but was limited to children under 20 years of age.

The death rate from other types of cancer was 2·6 times greater than the national rate. No type of cancer other than leukaemia was responsible for more than one death. It is possible that Down's syndrome predisposes to the developmen of many or all types of cancer, but this cannot be concluded from the present data alone. If there is any predisposition to the development of other types of cancer, it is clearly less marked than the predisposition to leukaemia.

The parents of 1620 affected children have been followed from the time their child first attended The Hospital for Sick Children or were first notified to the local authority. The mortality among them from all causes was somewhat lower than would have been expected on the basis of the national death rates during the same period. Three deaths occurred from leukaemia against 1·2 expected and 57 deaths occurred from other types of cancer against 64 expected.

The three deaths from leukaemia all occurred in fathers of affected children and another father has died from leukaemia since the end of the follow-up period. A second case of leukaemia and a second case of Down's syndrome occurred in one family; but no chromosome abnormalities were detected in the 5 other members of the family examined.

We are most grateful to Dr. A. Elliott, Medical Officer of Health, Kent County Council, Dr. C. Bennett, Mental Health Department, Middlesex County Council, Dr. D. Magrath, Botley's Park Hospital, Dr. E. F. Hewlitt and Dr. E. W. Shepperd, Leavesden Hospital, Dr. G. S. Mansell, Leybourne Grange Colony, Dr. B. Matheson, South Ockenden Institution for Mental Defectives and Dr. W. H. K. Carpenter, Stoke Park Hospital for access to their records and for tracing patients or their relatives. We are grateful also to many members of the local authority staff, and to Miss Flora Callaby and Mrs. K. Evans for much of the detailed work involved in the follow-up, to Mr. H. Whitfield for computing the numbers of expected deaths on the London University Computer Unit's Mercury, and to the Medical Research Council's Clinical Effects of Radiation Research Unit, Edinburgh, for the chromosome studies.

### REFERENCES

Allen, G. and 18 co-signatories—(1961) *Lancet*, i, 775.
Baikie, A. G., Jacob, P. A., McBride, J. A. and Tough, I. M.—(1961) *Brit. med. J.*, i, 1564.
Bernard, J., Nathe, G., Delorme, J. C. and Barnoud, O.—(1955) *Arch. franc. Pediat.*, **12**, 470.
Buckton, K. E., Harnden, D. G., Baikie, A. G. and Woods; A. E.—(1961) *Lancet*, i, 171.
Carter, C. O.—(1958) *J. ment. Def. Res.*, **2**, 64.
Court Brown, W. M. and Doll, R.—(1961) *Brit. med. J.*, i, 981.
Ford, C. E., Jones, K. W., Miller, O. J., Mittwoch, U., Penrose, L. S., Ridler, M. and Shapiro, A.—(1959) *Lancet*, i, 709.
Jacobs, P. A., Baikie, A. G., Court Brown, W. M. and Strong, J. A.—(1959) *Ibid.*, i, 710.
Krivit, W. and Good, R. A.—(1957) *Amer. J. Dis. Child.*, **94**, 289.
Lejeune, J., Gauthier, M. and Turpin, R.—(1959) *C.R. Acad. Sci., Paris*, **248**, 602.
Miller, O. J., Breg, N. R., Schmickel, R. D. and Tretter, W.—(1961) *Lancet*, ii, 78.
Penrose, L. S.—(1932) *J. Genet.*, **25**, 407.—(1933) *Ibid.*, **27**, 219.
Record, R. G. and Smith, A.—(1955) *Brit. J. prev. soc. Med.*, **9**, 10.
Stewart, A., Webb, J. and Hewitt, D.—(1958) *Brit. med. J.*, i, 1495.
Wald, N., Borges, W. H., Li, C. C., Turner, J. H. and Harnois, M. C.—(1961) *Lancet*, i, 1228.